# Reactive Oxygen Species-Responsive Miktoarm Amphiphile for Triggered Intracellular Release of Anti-Cancer Therapeutics

**DOI:** 10.3390/polym13244418

**Published:** 2021-12-16

**Authors:** Hyun-Chul Kim, Eunjoo Kim, Se Guen Lee, Sung Jun Lee, Sang Won Jeong, Young Jae Lee, Mi Kyung Kwon, Seong-Kyoon Choi, Jun Seong Hwang, Eunsook Choi

**Affiliations:** 1 Division of Biotechnology, Daegu Gyeongbuk Institute of Science and Technology (DGIST), Daegu 42988, Korea; sklee@dgist.ac.kr (S.G.L.); schrisj@dgist.ac.kr (S.J.L.); sjeong@dgist.ac.kr (S.W.J.); yj.lee@dgist.ac.kr (Y.J.L.); nodamenote@dgist.ac.kr (M.K.K.); cskbest@dgist.ac.kr (S.-K.C.); romantic333@dgist.ac.kr (J.S.H.); 2 Division of Electronics and Information Systems, Daegu Gyeongbuk Institute of Science and Technology (DGIST), Daegu 42988, Korea; ejkim@dgist.ac.kr

**Keywords:** drug delivery system, miktoarm amphiphile, ROS-responsive, tocopheryl derivate, tumor therapy

## Abstract

Reactive oxygen species (ROS)-responsive nanocarriers have received considerable research attention as putative cancer treatments because their tumor cell targets have high ROS levels. Here, we synthesized a miktoarm amphiphile of dithioketal-linked ditocopheryl polyethylene glycol (DTTP) by introducing ROS-cleavable thioketal groups as linkers between the hydrophilic and hydrophobic moieties. We used the product as a carrier for the controlled release of doxorubicin (DOX). DTTP has a critical micelle concentration (CMC) as low as 1.55 μg/mL (4.18 × 10^−4^ mM), encapsulation efficiency as high as 43.6 ± 0.23% and 14.6 nm particle size. The DTTP micelles were very responsive to ROS and released their DOX loads in a controlled manner. The tocopheryl derivates linked to DTTP generated ROS and added to the intracellular ROS in MCF-7 cancer cells but not in HEK-293 normal cells. In vitro cytotoxicity assays demonstrated that DOX-encapsulated DTTP micelles displayed strong antitumor activity but only slightly increased apoptosis in normal cells. This ROS-triggered, self-accelerating drug release device has high therapeutic efficacy and could be a practical new strategy for the clinical application of ROS-responsive drug delivery systems.

## 1. Introduction

Polymeric micelles are self-assembled core-shell structures. They consist of amphiphilic polymers with distinct hydrophilic and hydrophobic blocks and generally form in aqueous media. The self-assembly characteristic provides micelles of suitable size, low critical micelle concentration (CMC) and slow dissociation rate [1,2]. Polymeric micelles have been extensively investigated as anticancer drug delivery vehicles. Hydrophobic drugs are soluble in their hydrophobic cores. They can passively target tumor tissues through enhanced permeability and retention (EPR). They also have prolonged circulation time [3,4,5]. As drug delivery vehicles, polymeric micelles have the low CMC required for extended circulation in biological environments. Based on the dimensions of the physiological pores in the vasculature, the micelles must be smaller than 200 nm. Smaller micelles have better blood circulation, tissue penetration and cellular uptake than larger particles [6,7,8,9,10,11,12]. An effective approach towards improving polymer-based drug delivery is to adjust polymeric backbone architecture. Miktoarms are asymmetric star polymers composed of three or more branching strands emanating from a common core [13,14,15]. The properties of miktoarm polymers are superior to those of their linear diblock copolymer counterparts. The former has very low CMC, small size and the ability to encapsulate large amounts of drug molecules in aqueous self-assembly [15,16,17,18,19,20,21]. Moreover, miktoarm polymers have multiple branching segments that facilitate the incorporation of various stimulus-responsive units and biological targeting moieties. Hence, integrating them with units responding to various stimuli such as pH, redox, UV and temperature enables them to deliver different drugs to target sites [22,23,24,25,26,27,28,29,30,31].

In the present study, we report reactive oxygen species (ROS)-responsive micelles of a novel miktoarm amphiphile comprising hydrophilic poly(ethylene glycol) (PEG) and hydrophobic tocopheryl derivatives. ROS are widely distributed in living organisms and participate in the regulation of cell signaling pathways, the modulation of protein functions and the mediation of inflammation [32,33,34]. ROS concentrations are substantially higher in cancer than normal cells (≤100 × 10^−6^ M and ~20 × 10^−9^ M, respectively). The former has relatively higher metabolic rates and undergo oncogenesis [35,36]. One drawback of conventionally designed micellar nanocarriers is slow release of the drug cargo. Thioketal linkages can be rapidly cleaved by the relatively high intracellular ROS levels in the tumor microenvironment (TME) and degraded into acetone and thiols [37,38]. Hence, we inserted ROS-cleavable thioketals as linkers between the hydrophilic and hydrophobic moieties to improve drug release kinetics. Tocopheryl derivatives are vitamin E analogs that have anticancer and antitumor efficacy. They can destroy breast, lung and prostate cancer cells but are largely nontoxic to adjacent normal and paracancer cells [39]. Here, we used modified tocopheryl chains as the hydrophobic moieties because tocopheryl derivatives are hydrophobic and have anticancer efficacy. Tocopheryl derivatives can also rapidly generate intracellular ROS by interacting with mitochondrial respiratory complex Ⅱ and interfering with the mitochondrial ETC [40,41,42]. Thus, intracellular ROS is self-regenerated and amplified, which, in turn, facilitates the cleavage of thioketal linkages, destabilizes the micelles and releases the tocopheryl derivates and the drugs encapsulated in the micelles.

Here, we constructed an amphiphilic nanomaterial as an anticancer drug delivery platform containing a molecule generating ROS in cancer cells and thioketals that can be broken by the ROS. We synthesized a miktoarm amphiphile of dithioketal-linked ditocopheryl polyethylene glycol (named as DTTP) and confirmed its potential as an effective drug (DOX) carrier after ROS generation. We characterized the physicochemical properties of the micelles and evaluated the release of DOX from DOX-encapsulated DTTP micelles subjected to different ROS concentrations. We assessed the relative effects of DTTP micelle-mediated ROS generation on MCF-7 cancer and HEK-293 normal cells. Using the foregoing cells, we performed in vitro cytotoxicity assays on blank- and DOX-loaded DTTP micelles and compared them against that of HCl∙DOX solution

## 2. Materials and Methods

### 2.1. Materials

Methoxypoly (ethylene glycol)-amine (mPEG-NH_2_) with a molecular weight of 2000 g/mol was purchased from JenKem Technology (Plano, TX, USA). *N*-hydroxy succinimide (NHS), 4-dimethylaminopyridine (DMAP), pyrene, mercaptopropionic acid, ethylenediamine, dicyclohexylcarbodiimide (DCC), sodium dodecyl sulfate (SDS) and piperidine were acquired from Sigma-Aldrich Corp. (St. Louis, MO, USA). D-α-tocopherol, *N*,*N*-*bis*[(9*H*-fluoren-9-ylmethoxy)carbonyl]-*L*-lysine (Fmoc-Lys(Fmoc)-OH) and 1-(3-dimethylaminopropyl)-3-ethylcarbodiimide hydrochloride (EDC) were obtained from TCI (Portland, OR, USA). Doxorubicin hydrochloride (DOX∙HCl) was procured from Lancrix Chemicals (Shanghai, China). All solvents used in this experiment were reagent grade. MCF-7 and HEK-293 cells were bought from the Korean Cell Line Bank (Seoul, Korea). RPMI-1640, fetal bovine serum (FBS) and trypsin-EDTA (0.25%) were purchased from HyClone Laboratories (Logan, UT, USA). The cell viability assay (CCK-8) kit was acquired from Donginbiotech Co. (Seoul, Korea).

### 2.2. Synthesis

#### 2.2.1. Dipropionic Thioketal (DT)

Thioketal was synthesized according to a previously reported method, with a few modifications [36]. Briefly, 3.5 mL acetone was mixed with 8 mL mercaptopropionic acid and continuously stirred at 65 °C for 72 h. The mixture was then decanted into ice water to produce a white precipitate that was repeatedly washed with ice water and freeze-dried to obtain 8.4 g thioketal. The product was analyzed by ^1^H NMR (d^6^-DMSO, 400 MHz) spectroscopy and the following peaks were observed: *δ* (ppm) = 12.30 (s, 2H), 2.73 (t, 4H), 2.51(t, 4H) and 1.53 (s, 6H).

#### 2.2.2. Tocopheryl N-Hydrosuccinimide Ester (T-NHS)

*N*-hydroxysuccinimide (0.65 g; 6.78 mmol) and EDC (1.62 g; 8.50 mmol) were added to α-tocopheryl succinate solution (3.0 g; 5.65 mmol) in 30 mL methylene chloride. The mixture was stirred at room temperature for 24 h, washed with distilled water, dried with MgSO_4_ and concentrated under vacuum to generate 3.36 g tocopheryl-NHS as a white solid. The yield was 92.3%. The product was analyzed by ^1^H NMR (CDCl_3_, 400 MHz) spectroscopy and the following peaks were observed: δ (ppm) = 3.14 (m, 2H), 3.06 (m, 2H), 2.86 (s, 4H), 2.60 (t, 2H), 2.11 (s, 3H), 2.03 (s, 3H), 1.99 (s, 3H), 1.78 (d, 2H), 1.56 (m, 3H), 1.41 (m, 4H), 1.33 (m, 10H), 1.15 (m, 8H) and 0.88 (m, 12H).

#### 2.2.3. Tocopherylamine (TA)

Tocopheryl-NHS (2.0 g; 3.19 mmol) was gradually added to a solution of ethylenediamine (2.0 mL, 30.0 mmol) in 50 mL methylene chloride. The mixture was stirred at room temperature for 5 h, washed with distilled water, dried with MgSO_4_ and concentrated under vacuum to generate 1.64 g tocopherylamine as a yellow solid. The yield was 92.1%. The product was analyzed by ^1^H NMR (CDCl_3_, 400 MHz) spectroscopy and the following peaks were observed: *δ* (ppm) = 6.13 (s, 1H), 3.34 (m, 2H), 3.06 (m, 2H), 2.83 (s, 2H), 2.67 (m, 4H), 2.11 (s, 3H), 2.03 (s, 3H), 1.99 (s, 3H), 1.78 (d, 2H), 1.56 (m, 3H), 1.41 (m, 4H), 1.33 (m, 10H), 1.15 (m, 8H) and 0.88 (m, 12H.)

#### 2.2.4. Dithioketal-Linked Polyethylene Glycol (DTP)

To synthesize mPEG-Lysine (mPEG-Lys), Fmoc-Lys(Fmoc)-OH was linked to mPEG-NH_2_ in the presence of DCC and DMAP in methylene chloride. The mixture was then filtered, purified and desiccated to obtain the white solid Fmoc-Lys(Fmoc)-linked mPEG. The Fmoc groups were then removed with piperidine in dimethylformamide (DMF). To synthesizes DTP, mPEG-Lys (2.0 g; 0.94 mmol) was gradually added to a mixture of dipropionic thioketal (4.75 g; 18.8 mmol), EDC (0.72 g; 3.76 mmol) and DMAP (0.01 g; 0.08 mmol) in methylene chloride (30 mL). The mixture was stirred at room temperature for 24 h and washed with distilled water, and the organic layer was separated and dried with MgSO_4_. The solvent was evaporated and the product was precipitated in an excess of diethyl ether. The precipitate was filtered and dried under vacuum to obtain 1.97 g DTP. The yield was 79.7%.

#### 2.2.5. Dithioketal-Linked Ditocopherol Polyethylene Glycol (DTTP)

DTP (1.50 g; 0.57 mmol), tocopherylamine (0.76 g; 1.37 mmol), EDC (0.44 g; 2.28 mmol) and DMAP (0.01 g; 0.08 mmol) were dissolved in methylene chloride (50 mL) and stirred for 24 h. The mixture was washed with water and the organic layer was separated and dried with MgSO_4_. The solvent was evaporated and the product was precipitated in an excess of diethyl ether. The precipitate was filtered and dried under vacuum to obtain 1.81 g DTTP. The yield was 88.2%.

### 2.3. Polymer Characterization

#### 2.3.1. Nuclear Magnetic Resonance (NMR) Spectroscopy and Molecular Weight

The polymer and modified tocopherol were confirmed by ^1^H NMR. The spectra were obtained with a Bruker spectrometer (AVANCE III 400; Bruker, Billerica, MA, USA) operating at 400 MHz to detect protons. CDCl_3_ or deuterated dimethyl sulfoxide (DMSO) was the solvent. The molecular weight and molecular weight distribution of the polymer were determined using gel permeation chromatography (GPC, 1514, Waters, Milford, MA, USA) equipped with a Waters 2414 refractive index detector and Waters Styragel HR columns.

#### 2.3.2. Critical Micellar Concentration (CMC)

DTTP micelles were prepared by solvent evaporation. Briefly, 10 mg DTTP was dissolved in 1 mL methylene chloride. Then 10 mL distilled water was added to the polymer solution and it was stirred for 6 h under a nitrogen stream to evaporate the methylene chloride. The remaining micelles with hydrophobic tocopherol cores were in aqueous solution. The CMC of DTTP micelles was determined using a pyrene fluorescence probe. The polymer concentration was in the range of 1 × 10^−4^ ~ 1.0 mg/mL. The pyrene concentration was fixed at 6 × 10^−7^ M. The prepared samples were incubated at 37 °C with stirring for ~36 h to equilibrate the pyrene partition between the water and the micelles. The fluorescence spectra were measured with a Cary Eclipse fluorescence spectrometer (Varian Medical Systems, Palo Alto, CA, USA) at emission wavelength = 390 nm. CMC was estimated from the inflection points of the intensity ratios I_337_/I_333_ at various concentrations.

### 2.4. Micelle Characterization

#### 2.4.1. Particle Size, Zeta Potential and Stability of Micelles

Sizes, size distributions and zeta potentials of the DTTP- and DOX-loaded DTTP micelles were measured by dynamic light scattering (DLS; Zetasizer Nano ZS; Malvern Instruments, Malvern, UK). The stability of DTTP micelles was estimated using sodium dodecyl sulfate (SDS) as a destabilizing agent based on the method used in previous study [43]. The effect of SDS on micelles was investigated by means of DLS. Equal volumes of the micelle solution (0.1 mg/mL) and an SDS solution (0.5 mg/mL) were mixed. In the presence of a-5-fold weight of SDS, the light scattered intensity was monitored at pre-determined time intervals.

#### 2.4.2. DOX-Loaded Micelle Preparation and Drug Loading Content and Efficiency

DOX-loaded micelles were prepared by solvent evaporation. Briefly, 2 mg DOX∙HCl was reacted with 2 mol triethylamine (equivalent to DOX) in methanol at room temperature for 2 h to obtain the DOX. The latter was then mixed with 10 mg DTTP in methylene chloride. Phosphate-buffered saline (PBS; 10 mL) was added to a solution containing DOX and DTTP and the mixture was stirred under a nitrogen stream to evaporate the organic solvent. The mixture was dialyzed (MW cutoff ~2 kDa) against PBS for 72 h to remove any unloaded DOX. The solution was then passed through a syringe filter (0.2 μm pore diameter) to remove large aggregates. To determine drug loading, DOX-loaded micelles were prepared using Mass_DOX_/Mass_mPEG-TK-TP_ = 0.2, 0.4, 0.6, 0.8 and 1.0. The same procedure was executed on predetermined quantities of DOX. The DOX content was calculated using the calibration curve plotted for the fluorescence spectral analysis. The drug loading content was calculated as the ratio of the quantity of DOX loaded in the micelles to the weight of the DOX-loaded micelles. Drug encapsulation efficiency was calculated as the ratio of the amount of drug encapsulated in the micelles to the mass of DOX added.

#### 2.4.3. ROS Responsiveness of DTTP Micelles

Cleavage of the thioketal linkages in DTTP exposed to ROS was evaluated using a previously reported method, with minor modifications [32]. The DTTP was incubated in deuterated DMSO supplemented with a mixture of 100 μM H_2_O_2_ and 1.6 μΜ CuCl_2_ at 37 °C for 24 h and structurally analyzed by ^1^H NMR spectroscopy. The DTTP- and DOX-loaded micelles were incubated in an aqueous solution containing 100 μM H_2_O_2_ and 1.6 μΜ CuCl_2_ at 37 °C for 24 h and size and zeta potential changes in the micelles were measured by DLS.

#### 2.4.4. In Vitro Drug Release Study

DOX release from DOX-loaded DTTP micelles was assessed for three solutions containing different H_2_O_2_:PBS (pH 7.4) concentration ratios, PBS (pH 7.4), 100 μM H_2_O_2_ and 1 mM H_2_O_2_. Aliquots of DOX-loaded DTTP micelles were introduced into a dialysis tube (MWCO: ~2 kDa). The release medium was shaken at 120 rpm and 37 °C At predetermined intervals, 3 mL samples were withdrawn from the release medium and each was replaced with 3 mL fresh medium. DOX concentrations were determined by fluorescence spectrometry at excitation wavelength = 485 nm and emission wavelength = 550 nm (Cary Eclipse; Agilent Technologies, Santa Clara, CA, USA).

#### 2.4.5. Cell Culture and Intracellular ROS Detection

Human breast cancer cells (MCF-7) and normal human embryonic kidney cells (HEK-293) were incubated in RPMI 1640 containing 10% (*v*/*v*) FBS and 1% (*w*/*v*) antibiotics (penicillin-streptomycin) at 37 °C under a humidified atmosphere containing 5% CO_2_. Dichlorofluorescein diacetate (DCFA-DA) was used as a probe to detect the intracellular ROS. The MCF-7 and HEK-293 cells were seeded in glass-bottom dishes at a density of 5 × 10^5^/well and incubated for 24 h. The cells were treated with various DTTP concentrations and the untreated cells served as the control. After 4 h incubation with DTTP, the cells were washed twice with PBS, the media were replaced with 10 μL DCFA-DA and incubation resumed at 37 °C for 30 min. All cells were observed by confocal laser scanning microscopy (CLSM; Olympus/PV1200; Olympus Corp., Tokyo, Japan). Among the CLSM images, the intensities of the ten brightest spots in the same area were determined by digitization with UN-SCAN-IT software (Silk Scientific, Inc., Provo, UT, USA). The digitalized intensities were recorded as means ± S.D.

#### 2.4.6. In Vitro Cytotoxicity and Cellular Uptake Studies

To determine DTTP micelle cytotoxicity, MCF-7 and HEK-293 cells were seeded at 5 × 10^5^/well in 96-well plates. Each well contained 100 μL medium and the cells were incubated for 24 h. The cells were incubated with 0–100 μg/mL micelles in PBS for 24 h. Cells cultured in PBS served as the control. A CCK-8 assay was used to evaluate the micelle cytotoxicity. CCK-8 reagent (10 μL) was added to each well and the cells were incubated for another 2 h. A microplate reader (Multiskan; Thermo Fisher Scientific, Waltham, MA, USA) read the absorbances at 450 nm. Cytotoxicity was expressed as % viable cells relative to control cell viability. All experiments were performed in triplicate and the data were recorded as means ± S.D. The MCF-7 and HEK-293 cells were evenly seeded into 96-well plates at 5 × 10^3^/well and incubated for 24 h. The DOX-loaded micelles were added to make up DOX concentrations of 0 μM, 0.2 μM, 0.5 μM, 1 μM, 2 μM and 5 μM. Incubation proceeded for 24 h and DOX-loaded micelle cytotoxicity was evaluated by the CCK-8 assay. Free DOX cytotoxicity was also tested. To visualize cellular uptake of DOX-loaded DTTP, MCF-7 cells were seeded in glass-bottom dishes at 1 × 10^5^/well and cultured for 24 h. All cells were then incubated with DOX-loaded DTTP containing 1.0 μg/mL DOX and with 1.0 μg/mL free DOX for 24 h. The media were then removed and the cells were washed with PBS to remove extracellular micelles. The nuclei were stained with 4′,6-diamidino-2-phenylindole (DAPI) for 10 min and the cells were observed under a CLSM.

## 3. Results and Discussion

### 3.1. DTTP Synthesis and Characterization

**Scheme 1:**
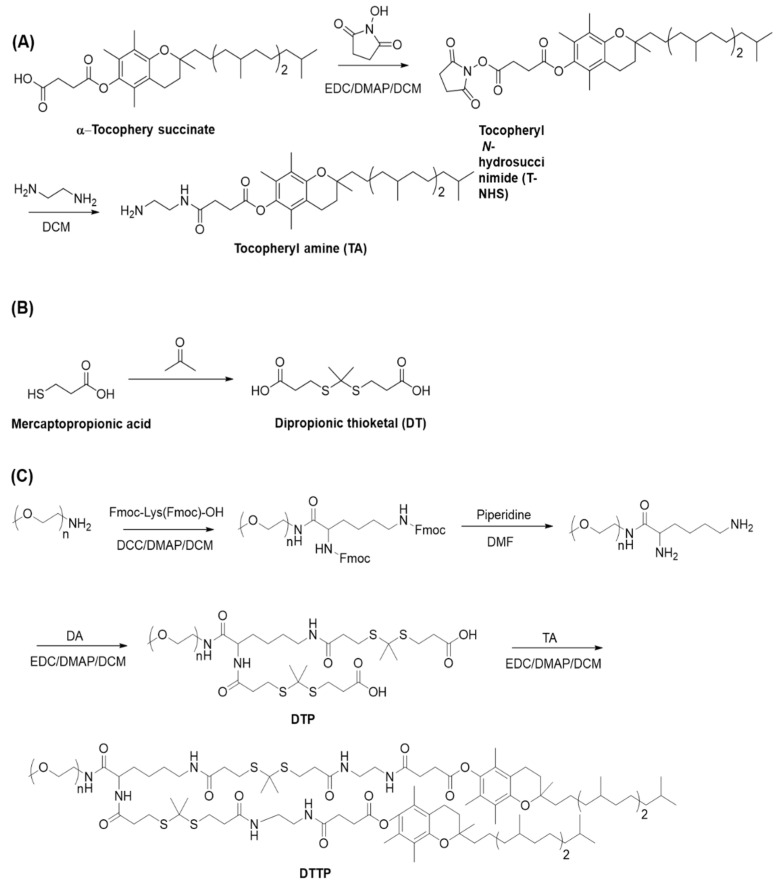
Syntheses of tocopherylamine (TA) (**A**), dipropionic thioketal (DT) (**B**) and dithioketal-linked ditocopherol polyethylene glycol (DTTP) (**C**).

We prepared an amphiphile containing ROS-cleavable thioketal as a connecting group between hydrophilic PEG and hydrophobic tocopherol units (Scheme 1). Dipropionic thioketal was prepared by reacting mercaptopropionic acid and acetone. The amine-functionalized tocopherol was synthesized by a two-step reaction.

**Figure 1:**
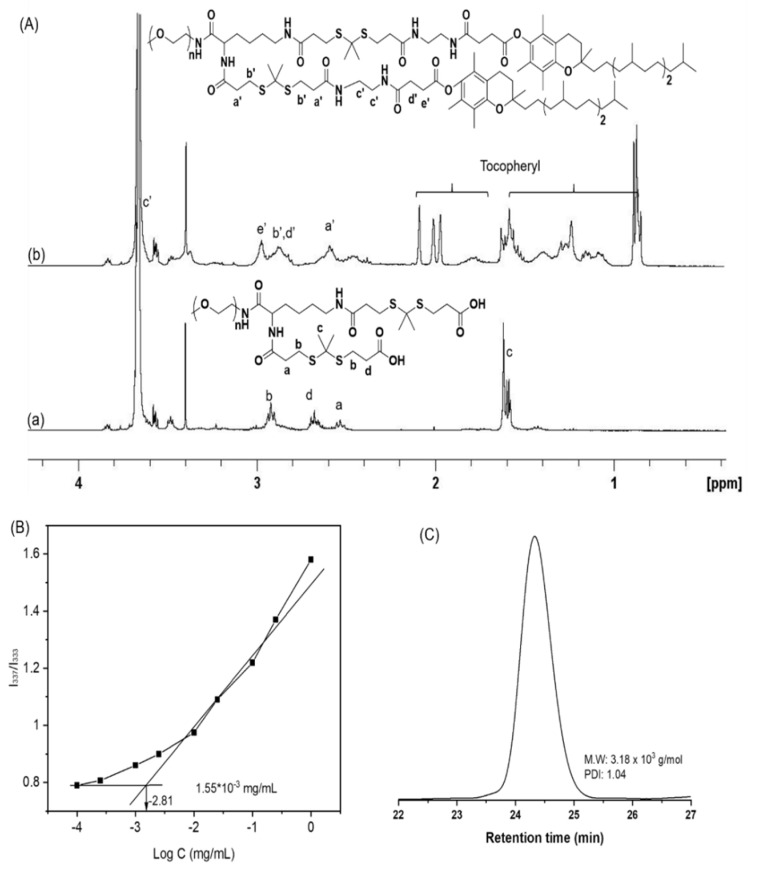
^1^H NMR spectra of dithioketal-linked polyethylene glycol (DTP) (a) and dithioketal-linked ditocopherol polyethylene glycol (DTTP) (b) in CDCl3 (**A**). Plot of intensity ratios I_337_/I_333_ from pyrene excitation spectra vs. logarithm of DTTP concentration (**B**). GPC trace of DTTP (**C**).

First, tocopherol succinimide ester was prepared by reacting tocopherol succinate and *N*-hydroxy succinimide using EDC as a coupling agent. Second, the tocopheryl succinimide ester was reacted with excess ethylenediamine to generate a tocopheryl derivate containing a terminal amine group. The DTTP was synthesized by a four-step reaction. Lysine linked-mPEG was synthesized by condensing mPEG-NH_2_ and Fmoc-Lys(Fmoc)-OH and deprotecting the Fmoc groups with piperidine. Dithioketal-linked mPEG (DTP) was synthesized by reacting the two amino groups in lysine-linked mPEG with excess dipropionic thioketal. The latter was amidated with the amine-functionalized tocopheryl derivate to generate the DTTP. The mPEG-TK-TP structure was confirmed by ^1^H NMR (Figure 1A). The chemical shifts at 3.41 ppm and 3.49–3.75 ppm were ascribed to the protons in the mPEG. The chemical shifts at 2.92 ppm, 2.68 ppm and 2.52 ppm were assigned to the methylene groups of thioketal (-CH_2_CH_2_S-, b, d, a). The strong resonance peak at 1.61 ppm was attributed to the methyl group (-S(CH_3_)C(CH_3_)S-, c). Hence, thioketal moieties were successfully attached to the two amino groups of lysine-linked mPEG (Figure 1A(a)). Comparison of the defined peak integrals of the protons in PEG (3.41 ppm) and the methyl group (1.61 ppm) in the thioketal indicated that the thioketal moieties introduced into the lysine-linked mPEG were nearly 100%. The amidation reaction between the two carboxylic groups in DTP and the amine-functionalized tocopheryl derivate was confirmed by ^1^H NMR. Characteristic peaks appeared at 2.97 ppm, 2.87 ppm and 2.59 ppm (e’, b’, d’ a’) (Figure 1(Ab) and disappeared at 2.92 ppm and 2.68 ppm (Figure 1A) and they corresponded to the methylene protons in the thioketal unit. Proton peaks below 2.5 ppm were ascribed to the tocopheryl. GPC measurement of DTTP was performed using tetrahydrofuran as a mobile phase. The number-average molecular weight of DTTP was 3.18 × 10^3^ g/mol with a PDI value of 1.04 determined using GPC with a polystyrene standard (Figure 1C). Considering the molecular weight of PEG and tocopheryl units, the theoretically calculated molecular weight was approximately 3.7 × 10^3^ g/mol and the discrepancy between the calculated molecular weight and GPC measurement molecular weight may be attributed to the difference in the hydrodynamic property between the DTTP and the polystyrene standard.

The amphiphilic DTTP consisted of hydrophilic PEG and two hydrophobic tocopherol units and self-assembled in the solution into nanometer aggregates. DTTP micelle formation was confirmed by fluorescence spectroscopy using pyrene as a probe. We plotted the fluorescence intensity ratio (I_337_/I_333_) as a function of the logarithm of the DTTP concentration (Figure 1B). We interpolated the CMC from the intersection between the baseline and the trend line. The CMC of DTTP was 1.55 μg/mL (4.18 × 10^−4^ mM) which was less those of TPGS2K (tocopheryl derivate linked with PEG; MW = 2K; 21.9 μg/mL) and TPGS1K (tocopheryl derivate linked with PEG; MW = 1K; 200 μg/mL). The latter are widely-used surfactants [44,45]. Compared with linear diblock copolymers, the DTTP had a lower CMC because of its greater hydrophobicity resulting from its two hydrophobic tocopheryl groups and the structural characteristics of the miktoarm polymer.

### 3.2. DTTP Nanoparticle Preparation and Characterization

**Figure 2:**
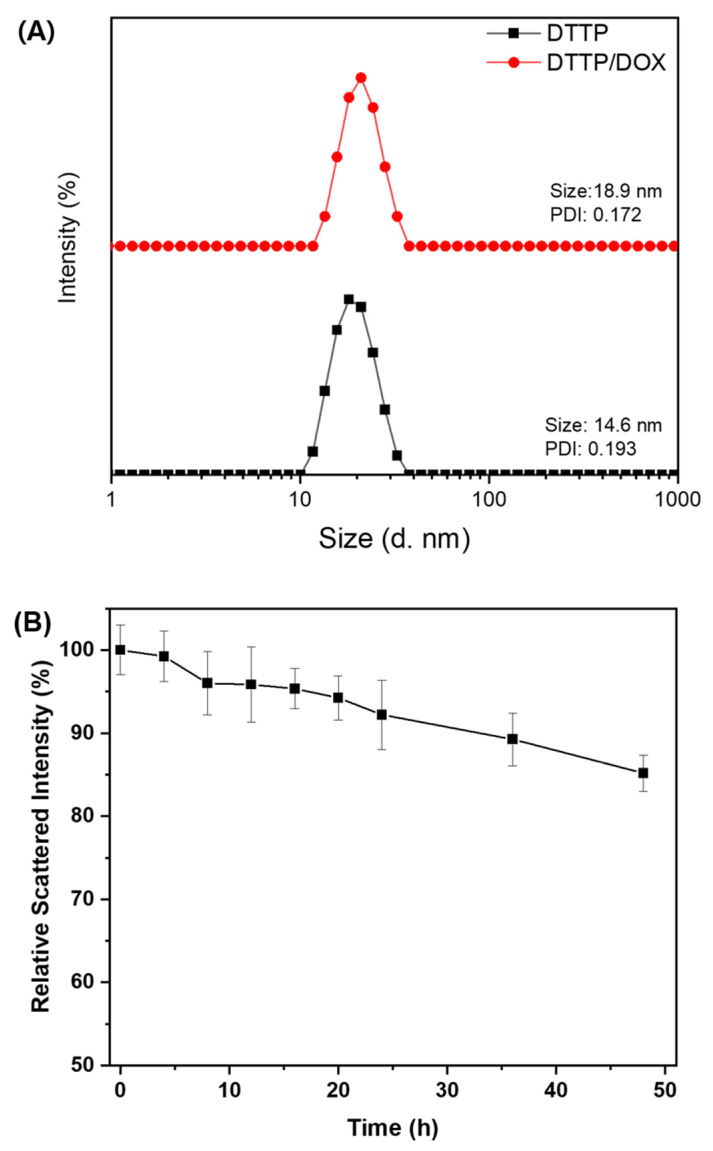
Particle size distributions of blank DTTP and DOX-loaded DTTP micelles (**A**). Scattered light intensity of DTTP micelles after addition of a 5-fold weight excess of SDS (Data are means ± S.D. of three independent experiments) (**B**).

DTTP self-assembled to form nanostructures in aqueous media because of the hydrophilic PEG and the hydrophobic tocopheryl groups in its molecular structure. Here, we applied the solvent evaporation method to prepare the DTTP nanoparticles. The drug-loading content and efficiency of the DTTP micelles were determined using DOX as a hydrophobic drug. The polymer:DOX feed ratios were in the range of 1:0.2–1:1 and the polymer concentration was 1 mg/mL. At a polymer:DOX feed ratio of 1:0.2, the maximum loading content and efficiency of the mPEG-TK-TP micelles were 8.52 ± 0.22% and 42.6 ± 2.3%, respectively. The DOX loading content and efficiency slightly decreased with increasing polymer:DOX feed ratio (Table 1). We used DLS to determine the size and distribution of the blank- and DOX-loaded micelles with maximum loading content and efficiency. The diameter of the DOX-loaded micelles was 18.9 nm. By contrast, the diameter of the DTTP micelles was 14.6 nm. Thus, the particle size changed in response to DOX encapsulation (Figure 2B). Moreover, the DOX-loaded micelle and DTTP particle sizes were within the desired 10–100-nm range. Therefore, these structures could avoid renal clearance and recognition by macrophages. The zeta potentials of the blank- and DOX-loaded micelles were 1.30 ± 3.91 and 1.88 ± 1.99 mV, respectively. For this reason, the neutrally charged PEG was localized mainly to the particle surfaces. To evaluate micelle stability, excess SDS as a destabilizing agent was added to the micelle solution and the scattered light intensity was monitored over time using DLS. When a DTTP micelle solution (0.1 mg/mL) was mixed with an equal volume of a concentrated SDS solution (0.5 mg/mL), the DTTP micelles showed enhanced stability as the scattered light intensity was reduced by less than 15% over 48 h. It is notable that, considering that DTTP is composed of weakly hydrophobic tocopheryl groups, the stability of DTTP micelles was comparable to that of the crosslinked micelles [46].

### 3.3. ROS Responsiveness of DTTP

**Figure 3:**
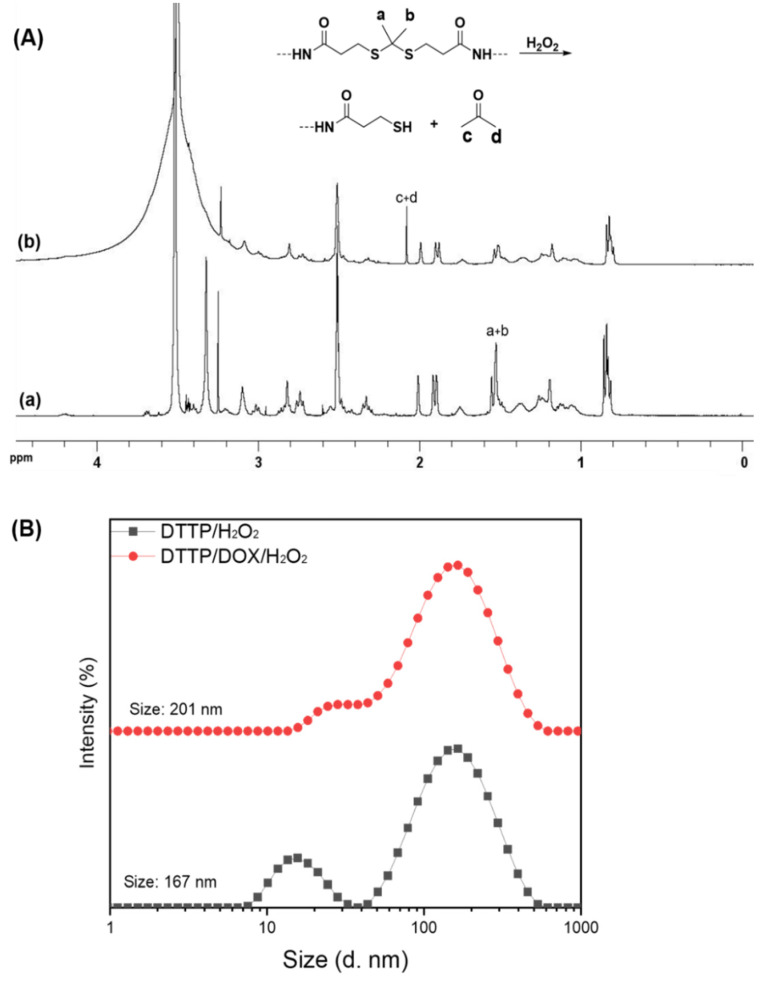
^1^H NMR spectra of DTTP (a) and cleaved DTTP in deuterated DMSO containing H_2_O_2_ and metal tracer (CuCl_2_) (b) (**A**). Particle size distributions of DTTP- and DOX-loaded micelles measured by DLS after incubation at 37 °C for 24 h in aqueous solutions containing H_2_O_2_ and CuCl_2_ (**B**).

We used ^1^H NMR spectroscopy to examine DTTP cleavage in the presence of ROS. The DTTP was incubated in deuterated DMSO containing H_2_O_2_ and trace amounts of transition metal cations to trigger ROS cleavage and simulate the ROS environment. The DTTP was immersed in the solvent for 24 h and the chemical shift of the methyl group corresponding to the thioketals (1.62 ppm) was distinctly weaker than that of the DTTP. A new peak was observed at 2.10 ppm. It was assigned to the acetone formed as a DTTP cleavage by-product (Figure 3A). Hence, the ROS efficiently cleaved the thioketal linkages in DTTP. We used DLS to monitor the changes in the DTTP micelle size distributions in the absence and presence of ROS. Figure 3B shows that the average micelle size increased from 14.6 nm to 167.0 nm and the size distribution was bimodal under the ROS environment. Aggregation occurred in response to the hydrophobic interactions among the insoluble tocopherol moieties after detachment of the hydrophilic PEG shells from the micelles in the presence of ROS. The zeta potentials of the DTTP micelles decreased from 1.30 ± 3.91 mV to −13.9 ± 3.11 mV after exposure to the ROS environment possibly because the thiols generated from the thioketals were decomposed by the ROS. Thus, cleavage of the thioketals in the DTTP micelles in response to the ROS destabilized and disintegrated the micellar aggregates. The results were similar for both the DOX-loaded and blank micelles. The drug encapsulated in the micelles may be released by destabilization following micellar shedding via ROS-mediated cleavage of the thioketal linkages.

### 3.4. In Vitro Drug Release

**Figure 4:**
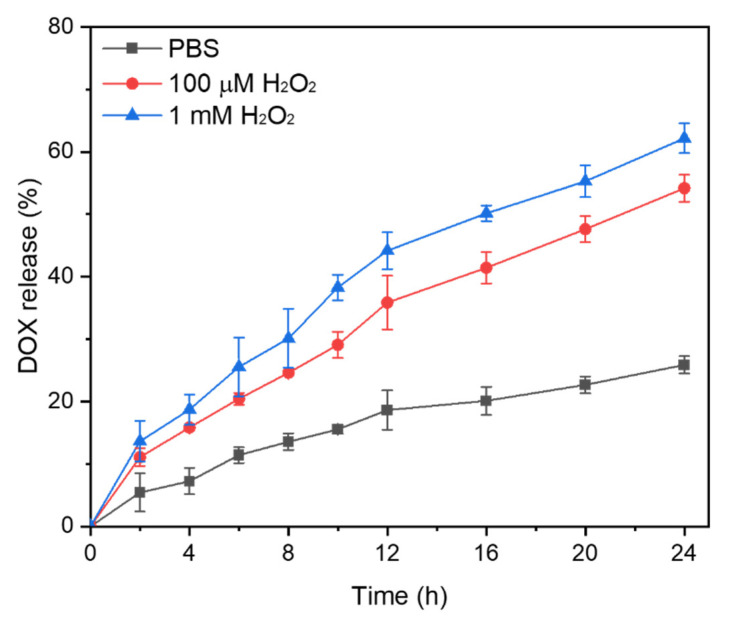
In vitro release profiles of DOX from DOX-loaded micelles subjected to different H_2_O_2_ concentrations. Data are means ± S.D. of three independent experiments.

Here, the thioketal linkers are broken by ROS stimulation and DOX is released from the micelles. To investigate the role of ROS in this system, we used H_2_O_2_ as the ROS stimulus in in vitro experiments. At predetermined time intervals over 24 h, we monitored the drug release profiles of DOX-loaded micelles subjected to various H_2_O_2_ concentrations (Figure 4). For the H_2_O_2_-free control, <26% of the drug load was released over 24 h. Thus, DOX entrapped in the hydrophobic micelle core was released by diffusion from the intact aggregates rather than by micellar disassembly. By contrast, 53% of the DOX was released after incubation with 100 μM H_2_O_2_ for 24 h. The rate of DOX release rose to 62% in response to exposure to 1 mM H_2_O_2_ for 24 h. Thus, increasing rates of thioketal linkage decomposition with H_2_O_2_ concentration had a strong and positive impact on the rate and cumulative amount of DOX release. As [ROS] > 100 μM in cancer cells, DOX-loaded DTTP micelles could provide controlled DOX release in them.

### 3.5. Intracellular ROS Detection

**Figure 5:**
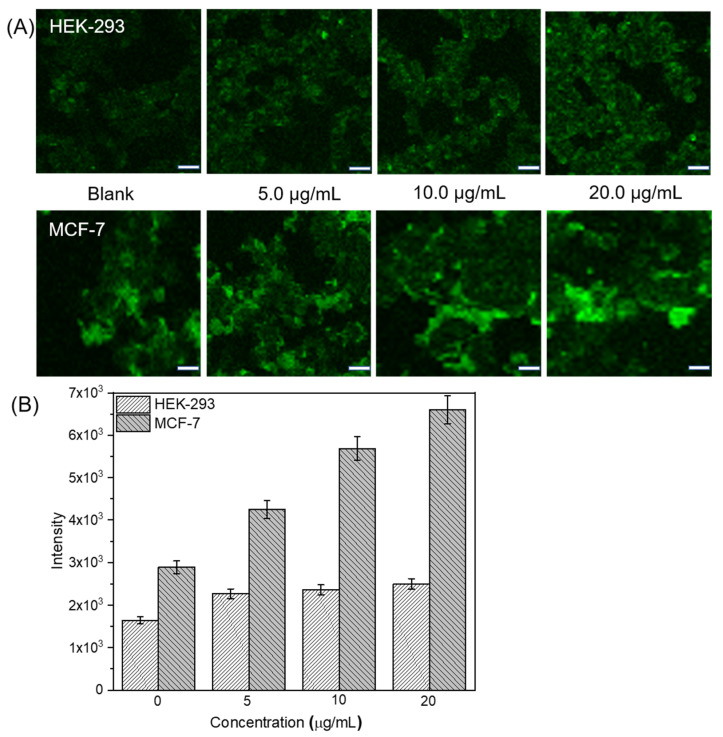
Evaluation of ROS-generating ability of DTTP micelles. CLSM images of HEK-293 normal and MCF-7 cancer cells treated with different DTTP micelle concentrations for 4 h (**A**). Scale bar: 20 μm. Quantitative UN-SCAN-IT analysis of CLSM images obtained for HEK-293 and MCF-7 cells treated with various DTTP micelle concentrations for 4 h (**B**).

ROS occur in nearly all cancers and participate in tumor development and progression. At moderate concentrations, ROS send vital survival and proliferation signals to cancer cells. At high concentrations, however, ROS induce cancer cell apoptosis or necrosis. In the present study, we empirically confirmed ROS generation by DTTP-containing tocopheryl derivatives and established whether the ROS also plays important roles in intracellular DOX release from drug-loaded DTTP micelles. We used DCFH-DA to evaluate ROS generation in MCF-7 cancer and HEK-293 normal cells. DCFH-DA is a non-fluorescent, cell-permeable probe that can be oxidized to dichlorofluorescein (DCF) which fluoresces green in the presence of intracellular ROS [32]. MCF-7 and HEK-293 cells were incubated with different DTTP micelle concentrations for 4 h, subjected to DCFH-DA for 30 min and viewed by confocal laser scanning microscopy (CLSM; Figure 5A). Green DCF fluorescence was strong in unstained MCF-7 cells but far weaker in HEK-293 cells. Hence, the intracellular ROS was high in the former. After DTTP treatment, the green fluorescence signal was stronger in the cancer than the normal cells. Comparison of normal cells against rapidly proliferating cancer cells may reveal relative differences in their ROS content that are attributable not to oncogenesis but rather to differences in their metabolic rates. MCF-7 cells incubated with DTTP fluoresced more strongly than those incubated without it. The tocopheryl derivate in DTTP can generate ROS. Moreover, green fluorescence in MCF-7 cells markedly increased with DTTP concentration. To quantitate ROS production by DTTP, we analyzed the CLSM images with UN-SCAN-IT gel software (Silk Scientific, Inc., Provo, UT, USA). The mean fluorescence intensities of the HEK-293 cells very slightly increased with DTTP concentration. By contrast, the mean fluorescence intensities of the MCF-7 cells dramatically increased with DTTP concentration. There was a 2.2-fold increase in the ROS levels of the MCF-7 cells treated with 20 μg/mL DTTP for 4 h (Figure 5B). Therefore, the DTTP tocopherol moieties generated ROS which added to the existing intracellular ROS.

### 3.6. Micelle Cytotoxicity

**Figure 6:**
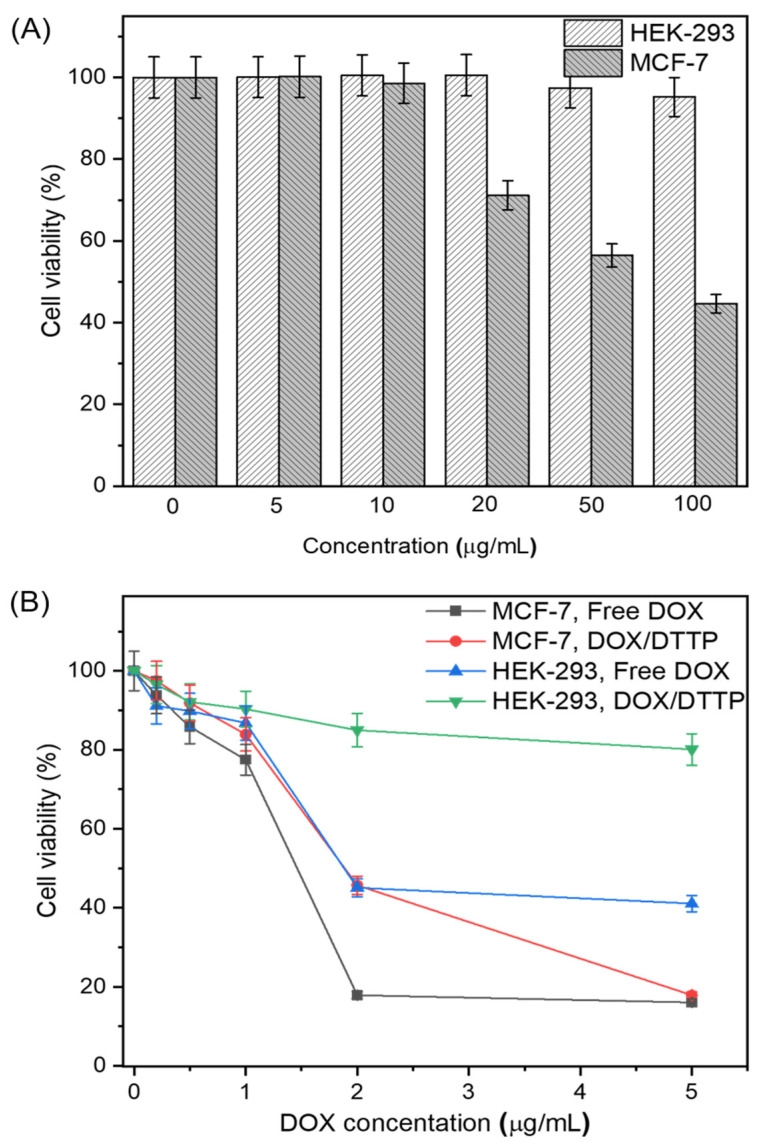
HEK-293 normal and MCF-7 cancer cell viability after incubation with different DTTP micelle concentrations for 24 h (**A**). HEK-293 and MCF-7 cell viability determined by CCK-8 assay after incubation with various concentrations of free DOX and DOX-loaded micelles for 24 h (**B**). Means ± S.D. of five independent experiments.

Cell viability in the presence of DTTP micelles was evaluated by the CCK-8 assay. Nanomaterial biosafety is a mandatory precondition for practical clinical application. We assessed the effects of blank DTTP micelles on MCF-7 and HEK-293 cell growth after 24 h of incubation. Figure 6A shows that the HEK-293 cell viability was higher than 90% even in the presence of 100 μg/mL DTTP micelles. Therefore, the latter exhibited low toxicity and stable drug delivery. At ≤10 μg/mL, DTTP micelles were not significantly cytotoxic to MCF-7 cells but were substantially cytotoxic at concentrations >20 μg/mL. Thus, DTTP micelles were considerably more toxic to MCF-7 than HEK-293 cells. The tocopheryl moieties desorbed from the micelles were absorbed by the cells after the ROS in the cancer cells cleaved the thioketal linkages in the DTTP micelles. We then measured the inhibitory effects of free DOX on MCF-7 and HEK-293 cells. There was significant dose-dependent cytotoxicity in both cell types incubated with DOX for 24 h. The IC_50_ were ~1.4 μg/mL and ~1.9 μg/mL for MCF-7 and HEK-293 cells, respectively (Figure 6B). We also investigated the viability of MCF-7 and HEK-293 cells exposed to DOX-loaded DTTP. HEK-293 viability remained >80% even at [DOX] = 5 μg/mL. For this reason, the DOX was tightly encapsulated in the DTTP micelles and there was negligible drug release under the very low ROS concentrations in normal cells. By contrast, DOX-loaded DTTP was evidently toxic to MCF-7 cells. The elevated ROS concentrations in the cancer cells cleaved the thioketal linkages in the DTTP micelles and the DOX was released and diffused. The foregoing findings demonstrate that ROS plays important roles in intracellular DOX release from DTTP micelles.

**Figure 7:**
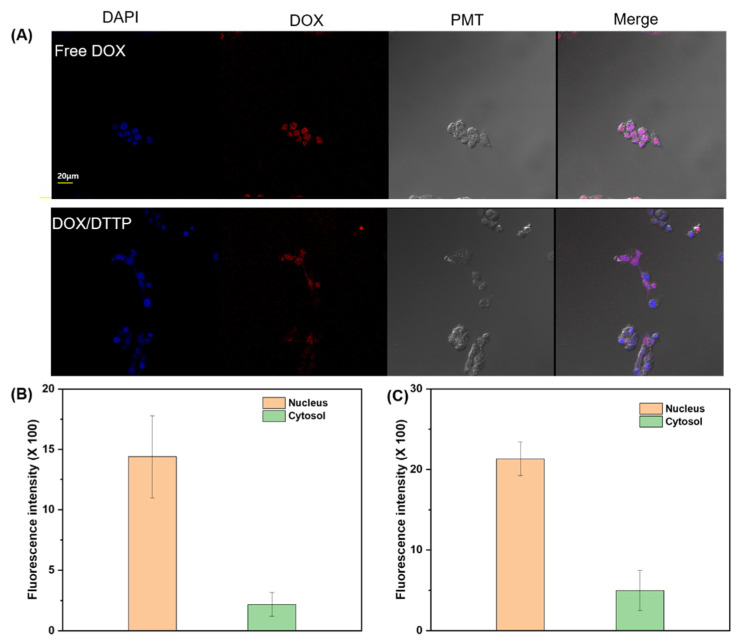
CLSM images of MCF-7 cells incubated with free DOX and DOX-loaded DTTP micelles for 24 h. Left to right: DAPI staining; intracellular DOX fluorescence; photomultiplier (PMT) images; and overlays of two images (**A**). Fluorescence intensity in nucleus and cytosol after treatment with free DOX (**B**). Fluorescence intensity in nucleus and cytosol after treatment with DOX-loaded micelles (**C**).

We also used CLSM to examine internalization and subcellular localization of free DOX and DOX-loaded micelles in MCF-7 cells. Figure 7A shows that the red fluorescence of free DOX localized mainly to the nuclei. DOX-loaded micelle fluorescence localized to both the nuclei and the cytoplasms. When the distribution of DOX in cells treated with DOX or DOX-loaded DTTP micelles was observed through fluorescence intensity, it was shown that the intensity of DOX was stronger in the nucleus than cytosol (Figure 7B,C). Therefore, the DOX was released intracellularly. As the DOX-loaded micelles were cytotoxic, they were effectively absorbed by the MCF-7 cells and their DOX loads were released in a controlled manner in response to intracellular ROS.

## 4. Conclusions

We successfully synthesized miktoarm amphiphiles that were joined by ROS-cleavable thioketal groups as linkers between hydrophilic PEG and hydrophobic tocopheryl units. They self-assembled to form stable colloidal micellar aggregates in aqueous solution. In the presence of ROS, the thioketal linkers were cleaved and destabilized the micelles. A cell viability study showed that the release of DOX from micelles loaded with it increased the ROS content inside MCF-7 cancer cells to a greater extent than it did in HEK-293 normal cells. The foregoing results suggest that ROS-responsive micelles are promising as anticancer drug carrier platforms with high antitumor efficacy.

## Figures and Tables

**Table 1:** DOX loading content and efficiency of DTTP micelles. Data are means ± S.D. of three independent experiments.

Micelles	Polymer:DOX Feed Weight Ratio	DOX Loading Content (wt.%)	DOX Loading Efficiency (%)
DTTP	1:0.2	8.52 ± 0.22	42.6 ± 0.23
	1:0.4	4.94 ± 0.29	12.4 ± 0.61
	1:0.6	4.23 ± 0.23	7.05 ± 0.30
	1:0.8	3.55 ± 0.32	4.44 ± 0.21
	1:1.0	3.13 ± 0.21	3.13 ± 0.15

## Data Availability

The data presented in this study are available upon request from the corresponding author.

## References

[1] Savi’c R., Azzam T., Eisenberg A., Maysinger D. (2006). Assessment of the integrity of poly(caprolactone)-b-poly(ethyleneoxide) micelles under biological conditions: A fluorogenic-based approach. Langmuir.

[2] Kataoka K., Harada A., Nagasaki Y. (2001). Block copolymer micelles for drug delivery: Design, characterization and biological significance. Adv. Drug Deliv. Rev..

[3] Letchford K., Burt H.M. (2012). Copolymer micelles and nanospheres with different in vitro stability demonstrate similar paclitaxel pharmacokinetics. Mol. Pharm..

[4] Kim J.Y., Kim S., Pinal R., Park K. (2011). Hydrotropic polymer micelles as versatile vehicles for delivery of poorly water-soluble drugs. J. Control. Release.

[5] Adams M.L., Lavasanifar A., Kwon G.S. (2003). Amphiphilic block copolymers for drug delivery. J. Pharm. Sci..

[6] Fox M.E., Szoka F.C., Fréchet J.M.J. (2009). Soluble polymer carriers for the treatment of cancer: The importance of molecular architecture. Acc. Chem. Res..

[7] Venkatachalam M.A., Rennke H.G. (1978). The structural and molecular basis of glomerular filtration. Circul. Res..

[8] Jain R.K. (1987). Transport of molecules across tumor vasculature. Cancer Metast. Rev..

[9] Alexis F., Pridgen E., Molnar L.K., Farokhzad O.C. (2008). Factors affecting the clearance and biodistribution of polymeric nanoparticles. Mol. Pharm..

[10] Owens D.E., Peppas N.A. (2006). Opsonization, biodistribution, and pharmacokinetics of polymeric nanoparticles. Int. J. Pharm..

[11] Vonarbourg A., Passirani C., Saulnier P., Benoit J.P. (2006). Parameters influencing the stealthiness of colloidal drug delivery systems. Biomaterials.

[12] Moghimi S.M., Hunter A.C., Murray J.C. (2001). Long-circulating and target-specific nanoparticles: Theory to practice. Pharmacol. Rev..

[13] Hadjichristidis N. (1999). Synthesis of miktoarm star (μ-star) polymers. J. Polym. Sci. Part A Polym. Chem..

[14] Hadjichristidis N., Iatrou H., Pitsikalis M., Mays J. (2006). Macromolecular architectures by living and controlled/living polymerizations. Prog. Polym. Sci..

[15] Victor L., Ashok K. (2020). Miktoarm star polymers: Branched architectures in drug delivery. Pharmaceutics.

[16] Sharma A., Kakkar A. (2015). Designing dendrimer and miktoarm polymer based multi-tasking nanocarriers for efficient medical therapy. Molecules.

[17] Khanna K., Varshney S., Kakkar A. (2010). Miktoarm star polymers: Advances in synthesis, self-assembly, and applications. Polym. Chem..

[18] Pispas S., Hadjichristidis N., Potemkin I., Khokhlov A. (2000). Effect of architecture on the micellization properties of block copolymers: A_2_B miktoarm stars vs. AB diblocks. Macromolecules.

[19] Hadjichristidis N., Pitsikalis M., Iatrou H., Driva P., Sakellariou G., Chatzichristidi M., Matyjaszewski K., Möller M. (2012). 6.03-Polymers with star-related structures: Synthesis, properties, and applications. Polymer Science: A Comprehensive Reference.

[20] Soliman G.M., Sharma R., Choi A.O., Varshney S.K., Winnik F.M., Kakkar A.K., Maysinger D. (2010). Tailoring the efficacy of nimodipine drug delivery using nanocarriers based on A2B miktoarm star polymers. Biomaterials.

[21] Wais U., Liu J., He T., Zhang H. (2017). Micellar and emulsion-assisted drug delivery: Comparison of miktoarm star polymers and block copolymers. Miktoarm Star Polymers: From Basics of Branched Architecture to Synthesis, Self-Assembly and Applications.

[22] Van Butsele K.V., Fustin C.A., Gohy J.F., Jérôme R., Jérôme C. (2009). Self-assembly and pH-responsiveness of ABC miktoarm star terpolymers. Langmuir.

[23] Cajot S., van Butsele K., Paillard A., Passirani C., Garcion E., Benoit J.P., Varshney S.K., Jérôme C. (2012). Smart nanocarriers for pH-triggered targeting and release of hydrophobic drugs. Acta Biomater..

[24] Zhang Y., Chen M., Luo X., Zhang H., Liu C., Li H., Li X. (2015). Tuning multiple arms for camptothecin and folate conjugations on star-shaped copolymers to enhance glutathione-mediated intracellular drug delivery. Polym. Chem..

[25] Zhou Q.-H., Lin J., Li L.-D., Shang L. (2015). Biodegradable micelles self-assembled from miktoarm star block copolymers for MTX delivery. Colloid Polym. Sci..

[26] Blasco E., Schmidt B.V.K.J., Barner-Kowollik C., Piñol M., Oriol L. (2014). A novel photoresponsive azobenzene-containing miktoarm star polymer: Self-assembly and photoresponse properties. Macromolecules.

[27] Wei H., Zhang X., Cheng C., Cheng S.-X., Zhuo R.-X. (2007). Self-assembled, thermosensitive micelles of a star block copolymer based on PMMA and PNIPAAm for controlled drug delivery. Biomaterials.

[28] Zhu J., Liu Y., Xiao L., Zhou P. (2016). Temperature-Sensitive (BA)(AC)_2_ Miktoarm star diblock copolymer based on PMMA, PPEGMA, and PNIPAm. Macromol. Chem. Phys..

[29] Yang P., Zhu F., Zhang Z., Cheng Y., Wang Z., Li Y. (2021). Stimuli-responsive polydopamine-based smart materials. Chem. Soc. Rev..

[30] Yang P., Zhang S., Chen X., Liu X., Wang Z., Li Y. (2020). Recent developments in polydopamine flurescent nanomaterials. Mater. Horiz..

[31] Anna B.-S. (2021). Towards a new class of stimuli-responsive polymer-based materials –Recent advances and challenges. Appl. Surf. Sci. Adv..

[32] Trachootham D., Alexandre J., Huang P. (2009). Targeting cancer cells by ROS-mediated mechanisms: A radical therapeutic approach. Nat. Rev. Drug Discov..

[33] Song C.-C., Du F.-S., Li Z.-C. (2014). Oxidation-responsive polymers for biomedical applications. J. Mater. Chem. B.

[34] Schumacker P.T. (2015). Reactive Oxygen Species in Cancer: A Dance with the Devil. Cancer Cell..

[35] Hu J.-J., Lei Q., Peng M.-Y., Zheng D.-W., Chen Y.-X., Zhang X.-Z. (2017). A positive feedback strategy for enhanced chemotherapy based on ROS-triggered self-accelerating drug release nanosystem. Biomaterials.

[36] Chen B., Zhang Y., Ran R., Wang B., Qin F., Zhang T., Wan G., Chen H., Wang Y. (2019). Reactive oxygen species-responsive nanoparticles based on a thioketal-containing poly(*β*-amino ester) for combining photothermal/photodynamic therapy and chemotherapy. Polym. Chem..

[37] Shim M.S., Xia Y. (2013). A reactive oxygen species (ROS)-responsive polymer for safe, efficient, and targeted gene delivery in cancer cells. Angew. Chem. Int. Ed..

[38] Lv X., Zhu Y., Ghandehari H., Yu A., Wang Y. (2019). An ROS-responsive and self-accelerating drug release nanoplatform for overcoming multidrug resistance. Chem Commun..

[39] Constantina C., Andreas P., Andreas I.C. (2008). Vitamin E and cancer: An insight into anticancer activities of vitamin E isomers and analogs. Int. J. Cancer.

[40] Prasad K.N., Kumar B., Yan X.-D., Hanson A.J., Cole W.C. (2003). α-Tocopheryl Succinate, the Most Effective Form of Vitamin E for Adjuvant Cancer Treatment: A Review. J. Am. Coll. Nutr..

[41] Dong Y.-H., Guo Y.-H., Gu X.-B. (2009). Anticancer mechanisms of vitamin E succinate. Chin. J. Cancer.

[42] Wang X.-F., Dong L., Zhao Y., Tomasetti M., Wu K., Neuzil J. (2006). Vitamin E analogues as anticancer agents: Lessons from studies with α-tocopheryl succinate. Mol. Nutr. Food. Res..

[43] Kim H.-C., Kim E., Ha T.-L., Lee S.G., Lee S.J., Jeong S.W. (2017). Highly stable and reduction responsive micelles from a novel polymeric surfactant with a repeating disulfide-based gemini structure for efficient drug delivery. Polymer.

[44] Mi Y., Liu Y., Feng S.-S. (2011). Formulation of docetaxel by folic acid-conjugated D-α-tocopheryl polyethylene glycol succinate 2000 (Vitamin E TPGS2k) micelles for targeted and synergistic chemotherapy. Biomaterials.

[45] Wang J., Sun J., Chen Q., Gao Y., Li L., Li H., Leng D., Wang Y., Sun Y., Jing Y. (2012). Star-shape copolymer of lysin-linked di-tocopherol polyethylene glycol 2000 succinate for doxorubicin delivery with reversal of multidrug resistance. Biomaterials.

[46] Lee S.-Y., Kim S., Tyler J., Park K., Cheng J.-X. (2013). Blood-stable, tumor-adaptable disulfide bonded mPEG-(Cys)_4_-PDLLA micelles for chemotherapy. Biomaterials.

